# The influence of walking speed and effects of signal processing methods on the level of human gait regularity during treadmill walking

**DOI:** 10.1186/s13102-022-00600-4

**Published:** 2022-12-10

**Authors:** Anna Brachman, Grzegorz Sobota, Bogdan Bacik

**Affiliations:** Institute of Sport Sciences, Department of Biomechanics, The Jerzy Kukuczka Academy of Physical Education, 72a Mikolowska, Katowice, Poland

**Keywords:** Complexity, Regularity, Gait, Treadmill, Vertical ground reaction force, Center of pressure, Sample entropy

## Abstract

**Background:**

In recent years the use of sample entropy (SampEn) to evaluate the complexity of the locomotor system in human gait data has gained in popularity. However, it has been suggested that SampEn is sensitive to various input parameters and signal preprocessing methods. This study quantified the effects of different temporal and spatial normalization approaches and various lengths of the template vector (m) on SampEn calculations. The discriminatory ability of SampEn was studied by comparing two walking conditions.

**Methods:**

Twenty-three participants (seven males, 55.7 ± 8.5 years, 165.7 ± 7.9 cm, 80.5 ± 16.7 kg) walked on a treadmill with preferred (Vpref) and maximum (Vmax) speed. Data were segmented and resampled (SEGM), resampled and spatially normalized (NORM), resampled and detrended (ZERO).

**Results:**

For vertical ground reaction force (vGRF) and center of pressure in anterio-posterior direction (COPap), in both walking conditions, SampEn was generally sensitive to the vector length and not to the data processing, except for COPap in ZERO, m = 2, 4. For the COPml SampEn behaved oppositely, it was sensitive to preprocessing method and not to the m length. The regularity of COPap and vGRF in all processed signals increased in Vmax condition. For the COPml only two signals, WHOLE and ZERO, revealed increased complexity caused by more demanding walking conditions.

**Conclusions:**

SampEn was able to discriminate between different walking conditions in all analyzed variables, but not in all signals. Depending on evaluated variable, SampEn was susceptible in different way for the m level and processing method. Hence, these should be checked and selected for each variable independently. For future studies evaluating influence of walking velocity on COP and vGRF regularity during treadmill walking it is advised to use raw time series. Furthermore, to maintain template vector which represents biological relevance it is advised to detect highest frequencies present in analyzed signals and evaluate minimal time interval which can reflect change caused by response of a neuromuscular system. During evaluating treadmill walking measured with 100 Hz sampling frequency it is recommended to adopt m from 6 to 10, when average stride time is up to about 1 s.

**Supplementary Information:**

The online version contains supplementary material available at 10.1186/s13102-022-00600-4.

## Introduction

“Nonlinear dynamical analysis is a powerful approach to understanding biological systems” [1]. Variability of human movement is not defined only through the amount of variance (e.g., standard deviation) but also through evaluation of the temporal variations in the movement output. This approach considers fluctuations throughout the movement, for example through gait cycle, taking into account previous states of neuromuscular system, and as such provide insight into how behaviour unfolds [2, 3]. Furthermore, analysis of the variability existing in movement patterns, which can be generated as a motor control system response for changes in the task difficulty or neuromuscular disability, provides a window into understanding the sophisticated strategies used to regulate movement or insight into the neuromuscular status of the patient [4].

The use of entropy methods to evaluate the complexity of the system in human data has gained in popularity [5–7], though, they are associated with some methodological challenges. It is said that Sample Entropy (SampEn) can be a promising tool for the analysis of gait complexity [8–12]. However, before applying it for the signal regularity evaluation some methodological issues still need to be resolved. Previous authors concluded that around 2000 points of data are necessary for SampEn stabilization [6, 7, 13]. In gait analysis, generally, it is difficult to obtain so many data points (e.g. 2000 stride intervals) and when dealing with pathological populations it can be unfeasible [6]. For this reason, SampEn has been calculated with a smaller number of data points or with using a continuous signal [13]. Various forms of continuous data [2, 8, 10] have been used in complexity analysis, however, the usage of center of pressure (COP) signal during a steady-state, where many consecutive steps can be captured, is becoming more popular [9, 14–16]. The COP at a given moment in time is the location of resultant vertical ground reaction force vector. The position of the COP under foot directly reflect the neural control of muscle force for body stabilization [17, 18]. Recently, it has been shown that traditional linear COP characteristics (e.g. first and second moment statistics values), in some cases, may not be sensitive enough to reveal significant changes of postural control caused by aging or pathological conditions [19, 20]. COP fluctuations reveal a complex output signal of the neuromuscular system in which various sensorimotor processes are reflected. Hence, it has been suggested that non—linear analysis (e.g. sample entropy) of the COP signal can provide surplus information to conventional measures [17, 21].

It was shown that SampEn is sensitive to various input parameters [7, 9]. McCamley et al. [13] highlighted the need of exploring the effect on SampEn in continuous data when using values of vector length (*m*) greater than 3. Although, Ahmadi et al. [9] concluded that the m = 2 ∼ 6 and tolerance level (r) = 0.2 × SD would be the preferred combination for continuous signal, however, it was determined only for the COP in the mediolateral (ml) direction. Another methodological issue is that some authors advised eliminating any trend before making meaningful interpretations from the statistical calculations [9, 14]. In some cases temporal and spatial normalization [14, 22] to detrend the signal was used. Previous authors suggested that by normalizing data in COP parameters more information about intra-stride dynamical features are obtained [22]. However, with this approach, information on extreme values, which also influence an intra-stride variability in individual cycles, is lost. Thus, we proposed another detrending method that enables to retain this information in COP signal dynamics.

Considering the increasing use of SampEn in analyzing human gait continuous signals, it is crucial to examine how parameter selection and various signal preprocessing methods would affect the outcomes. It is unknown how changing the vector length would affect the SampEn calculated on continuous signal like COP in antero-posterior (ap) direction and vertical ground reaction force (vGRF).

Furthermore, most studies to assess the discriminatory ability of SampEn involved different age groups or implemented cognitive loading during walking. Although speed has a significant effect on spatiotemporal variability [3, 23] as well as measures of dynamical systems [3, 24, 25], reports on the influence of gait speed on the regularity of the vertical ground reaction force or COPap time series are scarce. A greater understanding of control variables COP and vGRF regularity during walking could provide clearer insight into system behavior in more demanding conditions (walking with higher speed).

Therefore, our aim was twofold. Firstly, to examine how various vector lengths and signal preprocessing methods would influence SampEn of COP signal and vGRF. Secondly, to investigate the discriminatory ability of SampEn by analyzing two walking conditions: preferred and maximum tolerated speed. We hypothesized that our detrending method would be more discriminative when compared to conventional spatio-temporal normalization while comparing different walking velocities.

## Methods

### Biological data

Data for analysis purposes were derived from an experiment conducted previously on twenty-three participants (for details see [26]. Seven males, the mean age was 55.7 ± 8.5 years, mean height was 165.7 ± 7.9 cm, mean weight was 80.5 ± 16.7 kg. The mean comfortable gait speed on the treadmill was 0.81 ± 0.1 m/s and the maximum tolerated speed was 1.3 ± 0.2 m/s.

### Procedures

Each participant walked barefoot in two walking conditions: for 30 s with preferred speed (Vpref) and then 30 s with maximum tolerated speed (Vmax) on the Zebris treadmill system with mounted pressure platform (FDM–T, Zebris Medical GmbH, Germany), a sampling rate of 100 Hz was adopted. For subsequent analysis three signals were used: center of pressure displacement in the mediolateral (COPml), anteroposterior directions (COPap) and resultant vertical ground reaction force (vGRF). Data were processed using custom MATLAB codes (Mathworks, Inc., Natick, MA).

### Data processing

The beginning and the end of the data taken for the analysis were marked based on the COP signal with the removal of the first and last steps.

Four signal types were analyzed and three methods of preprocessing were used:

- Raw time series (WHOLE),
- Segmentation and resampling (SEGM),
- Segmentation, resampling and normalization (NORM),
- Segmentation, resampling and zeroing (ZERO).

**Fig. 1:**
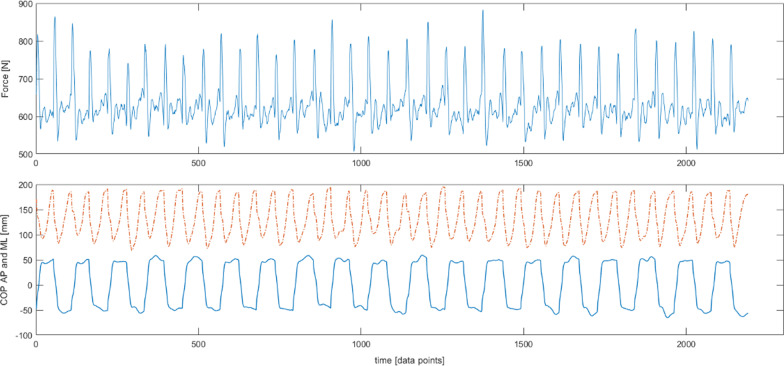
The raw time series. Trajectory of a COP in anterioposterior (AP—blue solid line) and mediolateral (ML—orange dotted line) direction and a resultant vertical ground reaction force (Force)

To obtain the same number of data points for raw time series the trials were limited to the lowest number of data points achieved by the subjects (2200). Raw time series (WHOLE) of ap/mlCOP and vGRF included the data without any amplitude or time base changes (Fig. 1).

#### Segmentation and resampling

**Fig. 2:**
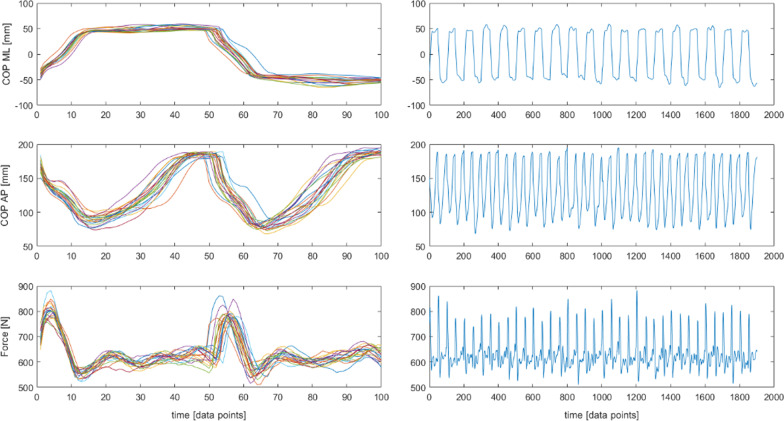
Segmented and resampled time series. Trajectory of a COP in anterioposterior (AP) and mediolateral (ML) direction and a resultant vertical ground reaction force (Force)

As data length and the number of data points within each stride can affect the outcome of SampEn analysis [6, 27], the trials were segmented into strides (the distance measured from the heel of the one foot to the heel of the same foot) and normalized in the temporal dimension in order to minimize differences due to a various subject’s walking speed [3, 9, 14, 22, 24]. Segmentation into strides was based on the vGRF signal indicating the beginning and the end of the single support phase of the one leg. Time intervals obtained determined the walking cycles for all variables. To acquire the same number of samples in each participant, in both walking conditions, signals were resampled to the mean number of data points per stride (100 samples per cycle). To obtain the same number of cycles in the entire study population in both walking conditions, the trials were limited to the lowest number of cycles achieved by the subjects (Fig. 2).

#### Segmentation, resampling and normalization

**Fig. 3:**
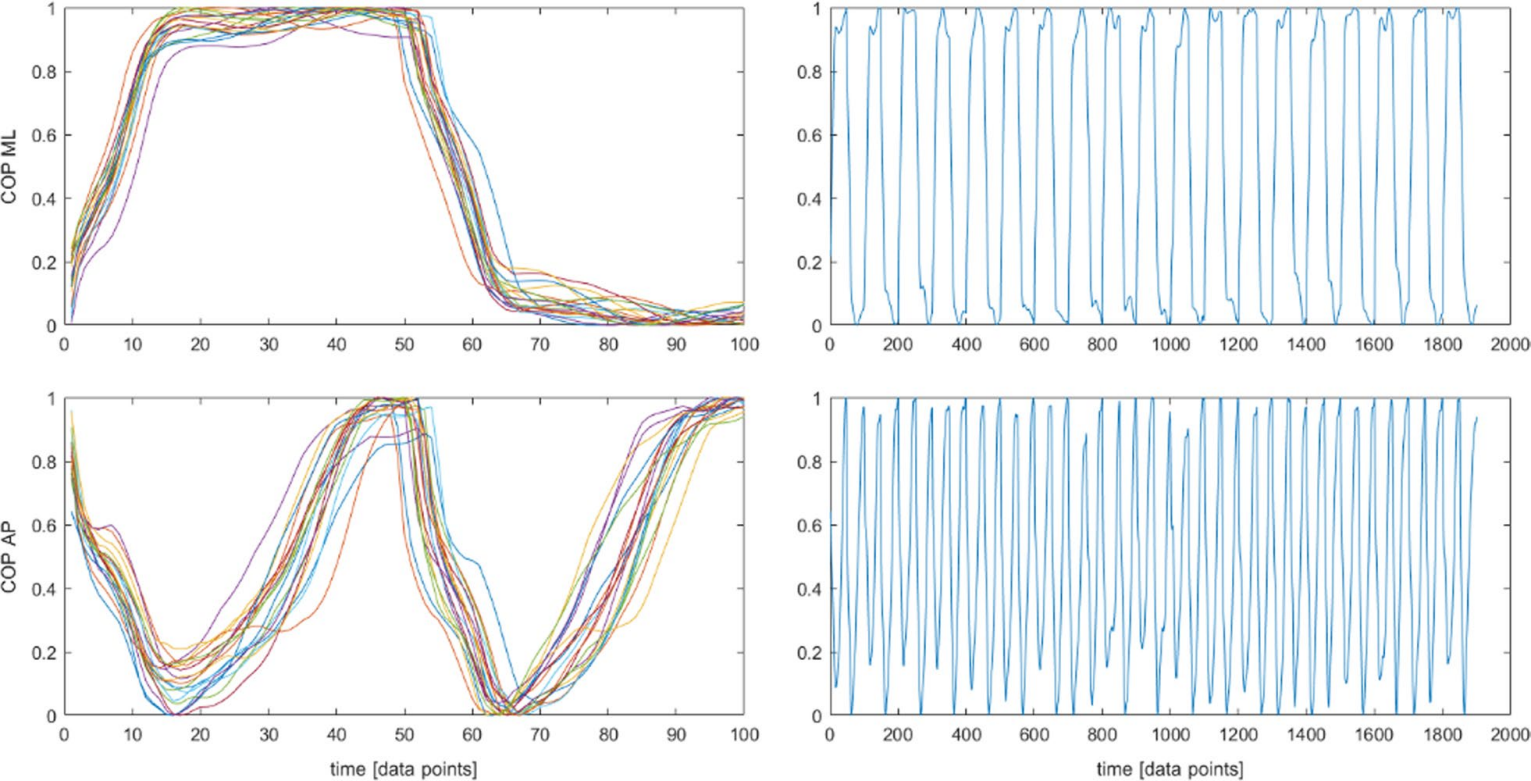
Segmented, spatially and temporally normalized time series. Trajectory of a COP in anterioposterior (AP) and mediolateral (ML) direction and a resultant vertical ground reaction force (Force)

To eliminate possible trend in the COP signal within the measurement area, after temporal normalization each stride was normalized in the spatial dimension so that the anterio-posterior signal of a single stride varied from  <0;1> of the stride length, the medio-lateral signal varied from <0;1> of the stride width [9, 14, 22, 27] (Fig. 3).

#### Segmentation, resampling and zeroing

**Fig. 4:**
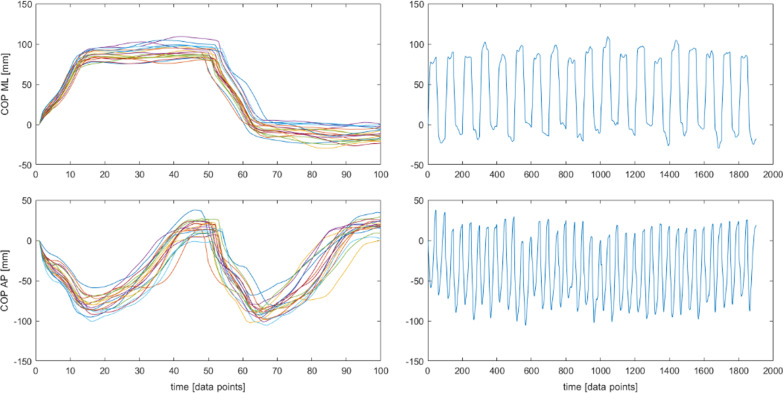
Segmented and detrended time series. Trajectory of a COP in anterioposterior (AP) and mediolateral (ML) direction and a resultant vertical ground reaction force (Force)

The raw data was initially processed as in SEGM, then, each subsequent cycle for the COP signal was starting at the coordinate (0, 0), i.e. as if the subject always started the cycle from the same point. All other values of the COPap/ml coordinates throughout the cycle were appropriately recalculated. This method enabled for the elimination of possible drift on the treadmill without losing absolute distances between data points (Fig. 4).

In the second part of the analysis, each of the time series was subjected to calculation of SampEn (m, r, N) based on the algorithm presented by Richman and Moorman [1]. SampEn has been defined as the negative natural logarithm for conditional probability that a series of data points within a certain distance, m, would be repeated within the distance m + 1.

$$SampEn\left( {m,r,N} \right) = - \ln \left[ {\frac{{A^{m + 1 } \left( r \right)}}{{B^{m} \left( r \right)}}} \right]$$

The N stands for the total number of data points in the time series, m represents the length of the vector that is compared during runs of data, and r stands for the sensitivity criterion in which alike vectors are considered similar. For the experimental data, m represented the number of samples that constituted a vector. The parameter r represented the tolerance of variance between samples. For human movement, a consistent or periodic gait pattern would elicit a low SampEn value and a more complex gait pattern (a time series with large differences between data points distances) would elicit a higher SampEn value. Thus, a perfectly repetitive time series gives SampEn value equaling 0 and a perfectly random time series gives a SampEn value converging toward infinity [5].

Sample entropy was calculated in two different walking conditions (Vpref, Vmax) for all signals in COPap and COPml (WHOLE, SEGM, NORM, ZERO) and for two signals in vGRF parameter (WHOLE, SEGM) using m = 2, 4, 6, 8, 10 and r as 0.2 of the average standard deviation of the time series [9, 14, 22]. The choice of r = 0.2 for our data was confirmed by method proposed by Lake et al. [28] and further statistical analysis for r ranged from 0.1 to 0.3 (Additional file 1:  Figs. A1–A9).

### Statistical analysis

A two-way ANOVA with HSD Tukey post–hoc was used to compare the effect of the m-level (m = 2, 4, 6, 8, 10) and data processing method (Type = WHOLE, SEGM, NORM, ZERO) on the calculated sample entropy. For the vertical ground reaction force ‘Type’ factor had two levels (WHOLE, SEGM). Each condition (Vpref, Vmax) was analyzed separately.

In the second part of the analysis, two-way mixed ANOVA (with m = 6 based on the results of the first part) with walking condition as a within-subjects and processing method as between-subjects factor was used to examine the effect of walking velocity on the signal regularity. In most cases data were normally distributed, additionally the maximum normalized residual test (Grubbs test) did not detect any outliers. In 9 for 100 subgroups the normality assumption was violated, however skewness was about │0.5│ with three exceptions. The F-test is said to be robust with respect to the assumption of normality and equality of variances so long as each group contains the same number of scores [29], however, if assumption of homogeneity was violated the correlations between means and variances were inspected. The assumption of sphericity was assessed using Mauchly’s test. When the assumption of uniformity was violated, an adjustment to the degrees of freedom of the F-ratio was made using Greenhouse–Geisser Epsilon, thereby making the F-test more conservative. Statistical analyses were carried out using Statistica software version 13.4, a p < 0.05 was considered significant.

## Results

### Walking with preferred speed

**Fig. 5:**
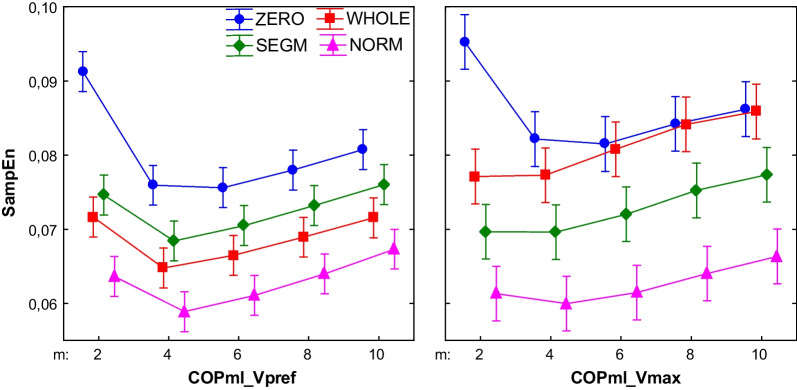
Effect of changing vector length (m) on the COPml SampEn for different processing methods (r = .02). (WHOLE) raw time series; (SEGM) segmented and resampled signal; (NORM) segmented, resampled and normalized signal; (ZERO) segmented, resampled and detrended signal; results for walking with preferred (Vpref) and maximum tolerated (Vmax) speed; the error bars indicate 0.95 confidence interval

**Fig. 6:**
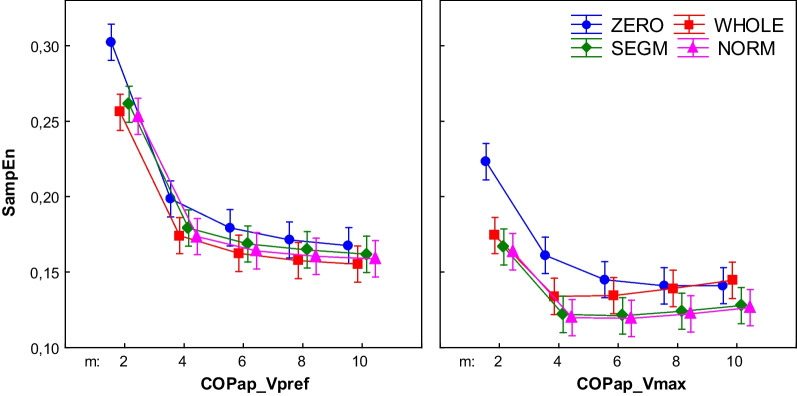
Effect of changing vector length (m) on the COPap SampEn for different processing methods (r = .02). (WHOLE) raw time series; (SEGM) segmented and resampled signal; (NORM) segmented, resampled and normalized signal; (ZERO) segmented, resampled and detrended signal; results for walking with preferred (Vpref) and maximum tolerated (Vmax) speed; the error bars indicate 0.95 confidence interval

**Fig. 7:**
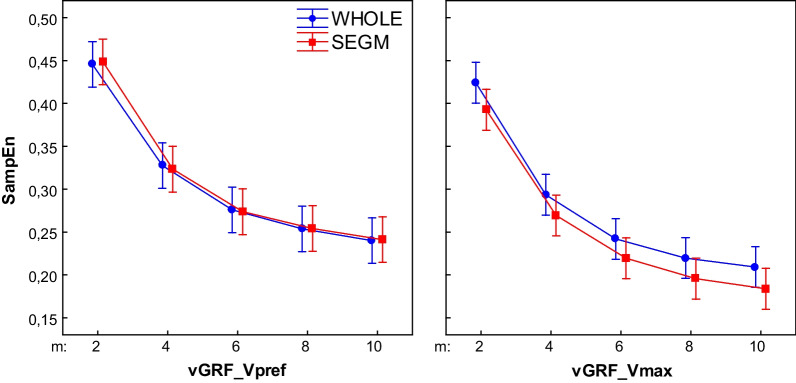
Effect of changing vector length (m) on the vGRF SampEn for different processing methods (r = .02). (WHOLE) raw time series; (SEGM) segmented and resampled signal; (NORM) segmented, resampled and normalized signal; (ZERO) segmented, resampled and detrended signal; results for walking with preferred (Vpref) and maximum tolerated (Vmax) speed; the error bars indicate 0.95 confidence interval

Generally, SampEn for the COPml signal was sensitive for different preprocessing methods, whereas for different m–level showed relative consistency. For the COPap and vGRF signals SampEn showed opposite dependency (Figs. 5, 6, 7).

Analyses revealed significant main effect of both Type and m parameter for COPml (Type: F3,440 = 141.14, p < 0.01; m: F4,440 = 26.35, p < 0.01) and COPap (Type: F3,440 = 16.33, p < 0.01; m: F4,440 = 240.33, p < 0.01). For a COPml also a significant interaction between Type and m was found (m*Type: F12,440 = 3.79, p < 0.01).

Post-hoc analysis revealed that COPml SampEn for Type = ZERO in m = 2, 4 was significantly higher in comparison with all signal types and in m = 6, 8, 10 it differed from Type = WHOLE, NORM. In contrast, SampEn for Type = NORM was significantly lower than for Type = ZERO, SEGM across all m levels and lower than for Type = WHOLE in m = 2. In Type = NORM and SEGM SampEn differed only between m = 4 and m = 10. In Type = WHOLE SampEn differed only between m = 2 and m = 4. In Type = ZERO only m = 2 was significantly higher than all other m levels.

For the COPap, in each preprocessed signal, entropy stabilized at m = 6. SampEn for Type = ZERO differed significantly from other signal types only in m = 2. Also only for m = 2 in all signal types SampEn values were significantly higher than for all other m levels.

For vGRF significant main effect was observed only for the m parameter (F4,220 = 77.54, p < 0.01). SampEn values did not differ between types across all m levels. Similarly to COPap, entropy stabilized at m = 6 in each signal type.

### Walking with maximum speed

Similarly as in Vpref, during walking with maximum speed SampEn for the COPml showed dependency of different preprocessing methods versus the COPap and vGRF parameters in which SampEn showed dependency of the m parameter. Analysis revealed significant main effect of both Type and m parameter for COPml (Type: F3,440 = 147.34, p < 0.01; m: F4,440 = 7.62, p < 0.01) and COPap (Type: F3,440 = 26.22, p < 0.01; m: F4,440 = 48.54, p < 0.01). Additionally in both COPml and COPap a significant interaction between Type and m was found (accordingly m*Type: F12,440 = 3.60, p < 0.01 and F12,440 = 2.51, p < 0.01).

For the COPml SampEn Type = ZERO in m = 2 was significantly higher than other signals. In m = 4, 6 it was significantly higher than for Type = SEGM, NORM and in m = 8, 10 it was higher than for Type = NORM. SampEn for Type = NORM was significantly lower than in all other types in all m levels, with one exception m = 2. Type WHOLE did not differ from Type = SEGM across all m levels. Generally, SampEn in all Types was stable across all m levels. Only Type = ZERO in m = 2 was significantly different from m = 4, 6, 8.

The COPap SampEn did not differ between all signal types in m = 6, 8, 10, except for Type = ZERO (m = 2, 4). In all types, SampEn was stable across all m levels, except m = 2.

For the vGRF significant main effect of both Type and m parameter was observed (Type: F4,220 = 11.19, p < 0.01; m: F4,220 = 102.79, p < 0.01). Both signal types did not differ across all m levels. In both signals, SampEn was significantly higher in m = 2 than all other m levels and m = 4 was significantly higher when compared to m = 8, 10.

### The comparison of walking conditions

Results showed that in the COPap as well as in the vGRF regularity increased with increasing velocity, whereas in the COPml only two of investigated signal types revealed decreased regularity.

For the COP parameters the results showed significant main effect of both Type and Velocity in COPml (Type: F3,88 = 27.46, p < 0.01; Velocity: F1,88 = 46.31, p < 0.01) and COPap (Type: F3,88 = 3.68, p < 0.02; Velocity: F1,88 = 364.4, p < 0.01) as well as interaction effect (accordingly Velocity*Type: F3,88 = 15.10 p < 0.01 and F3,88 = 4.54, p < 0.01).

**Fig. 8:**
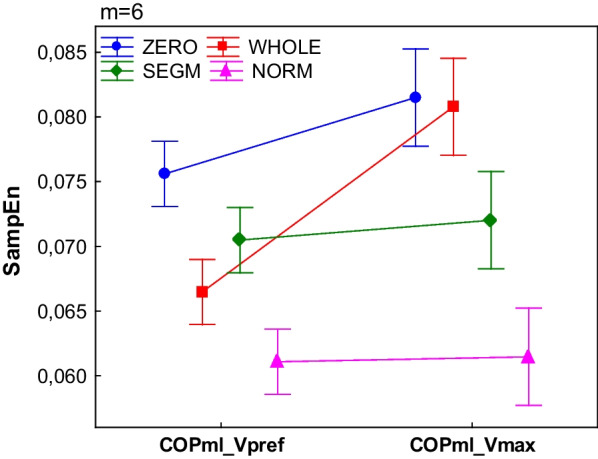
The effect of walking velocity on the regularity of the COPml for different processing methods. (WHOLE) raw time series; (SEGM) segmented and resampled signal; (NORM) segmented, resampled and normalized signal; (ZERO) segmented, resampled and detrended signal; SampEn input parameters m = 6, r = 0.2; the error bars indicate 0.95 confidence interval. Only WHOLE and ZERO signals showed significant differences between conditions (p < 0.05)

**Fig. 9:**
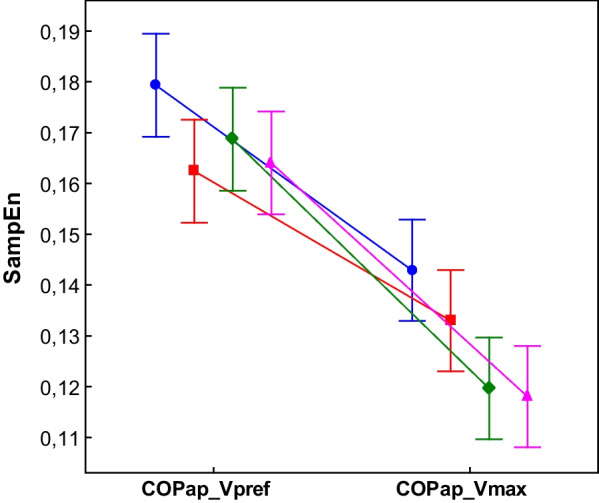
The effect of walking velocity on the regularity of the COPap for different processing methods. (WHOLE) raw time series; (SEGM) segmented and resampled signal; (NORM) segmented, resampled and normalized signal; (ZERO) segmented, resampled and detrended signal; SampEn input parameters m = 6, r = 0.2; the error bars indicate 0.95 confidence interval. All signals showed significant differences between conditions (p < 0.05)

The COPml SampEn in Types = SEGM, NORM did not change despite the change of walking conditions (Fig. 8). In Types = ZERO, WHOLE regularity decreased significantly in Vmax when compared to Vpref. In the Vpref condition, SampEn did not differ between Type = WHOLE and SEGM but in the Vmax condition, Type = WHOLE was significantly higher than Type = SEGM. For the COPap in the Vmax condition, regularity increased significantly for all investigated signal types (Fig. 9).

**Fig. 10:**
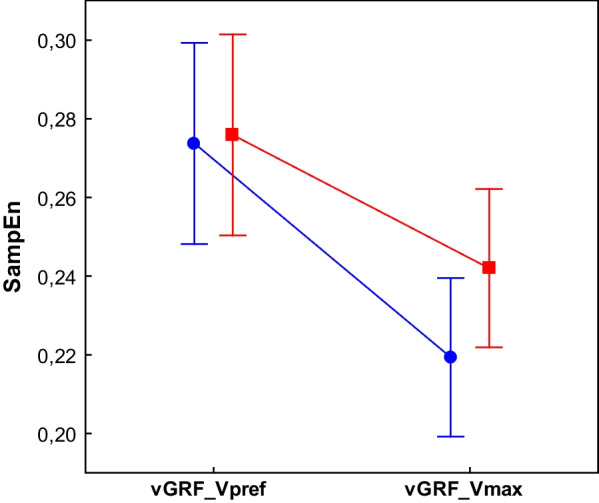
The effect of walking velocity on the regularity of the vGRF for different processing methods. (WHOLE) raw time series; (SEGM) segmented and resampled signal; SampEn input parameters m = 6, r = 0.2; the error bars indicate 0.95 confidence interval. Both signals revealed significant differences between conditions (p < 0.05)

For the vGRF the main effect of Velocity (F1,44 = 36.37, p < 0.01) was observed. For both signals, SampEn was significantly lower in the Vmax condition (Fig. 10).

## Discussion

The goal of this study was to identify the sensitivity of SampEn to variant values of parameter m and different preprocessing methods when applied to COPap, COPml and vGRF signals obtained during treadmill walking as well as SampEn sensitivity for changing walking velocity. One of general finding is that SampEn for the COPml signal was sensitive for different preprocessing methods, whereas for different m levels showed relative consistency. For the COPap and vGRF signals, SampEn showed opposite dependency. Furthermore, SampEn was able to discriminate between different walking conditions in all analyzed parameters.

During walking with preferred speed SampEn of vGRF of both preprocessed signals consistently decreased with increasing m. In previous studies [9, 11, 13] authors reported similar relationship for COP, joint angles and EEG signals. In our study the entropy of COPap in each preprocessed signal stabilized at m = 6. These results suggest that for vGRF and COPap during walking with preferred speed SampEn was sensitive to the length of the vector m and not to the data processing. A similar pattern could be observed in the Vmax condition.

Another observation is that SampEn for COPml parameter worked oppositely. Namely, it was sensitive to preprocessing method and not to the length of the m parameter, except for m = 2. Ahmadi et al. [9] reported decreasing trend of SampEn with increasing m in two out of six evaluated frequencies. In the current study sampling frequency was 100 Hz and each cycle contained 100 points, Ahmadi et al. [22] have resampled cycle to 142 points. In the literature, there are reports that SampEn is sensitive to the number of data points within the cycle [27], thus, we cannot directly compare our results to this obtained by Ahmadi et al. [9]. Nevertheless, we can observe that for f = 16 (which is equivalent to a frequency of 62 Hz) SampEn revealed very similar pattern like in our experiment and it plateaued at m = 4, however, SampEn value was higher than in our research. It seems to be consistent with findings reported previously—as the sampling frequency increases the spatial distance between data points decreases, which in turn, increases the number of vectors within each stride cycle and decreases SampEn value [27]. Interestingly, in the other work of Ahmadi et al. [22] authors used a sampling frequency of 60 Hz and obtained results similar to ours. Both parameters, COPml and COPap, as well as both signal types, whole and normalized data (120 samples per stride, m = 6, r = 0.2SD) yielded SampEn comparable to ours. Thus, there must have been another additional factor (than just a different frequency) that impacted the outcomes. In Ahmadi et al. [9] participants walked with higher velocity (1.0 m/s) than in the work of Ahmadi et al. [22] (0.8 m/s) and the current study (0.81 m/s SD 0.1). Different walking speeds could have influenced the subjects’ sensorimotor system and behavior [3, 24].

Another observation is that, for most of the m values, entropy for COPml for the NORM signal differed significantly from other signals. In both speeds, this signal gave the lowest SampEn values which implied that it was most regular. Previous authors [14, 22] suggested that signal should be detrended before calculation of sample entropy. The authors proposed spatio-temporal normalization (here: NORM) of the data and concluded that this method gives more information about intra-stride dynamical features. Furthermore, the authors [22] stated that normalized COPml signal is the best for showing the gait changes, also when compared to whole (raw) data. Current results are in contradiction to that finding. In our study, in the COPml spatially and temporally normalized signal SampEn showed no significant differences between Vpref and Vmax. A possible explanation for these discrepancies is that previous authors did not compare different walking speeds, but normal walking and walking with a dual-task. In the current study only two signals, WHOLE and ZERO, were sensitive enough to reveal changes in signal complexity caused by changes in walking velocity. Although spatio-temporal normalization removes trend, it additionally removes information on extreme values in individual cycles, which also contain information about intra-stride regularity. Thus, we proposed another detrending method (ZERO) which enabled to retain more data about signal dynamics. In the Vpref condition this signal was most complex, but in the Vmax condition entropy from the ZERO and WHOLE signal did not differ. The high SampEn during walking with a preferred speed could result from different spatial distances between the points. In the Vpref condition, distances were smaller than in Vmax. Hence, in the Vpref condition, the length between the last point in the cycle and the first point in the next cycle (which starts from point 0) might have decreased the regularity. Nevertheless, such signal processing allowed for differentiation of the signal complexity level between two different walking conditions and supported our hypothesis. Existing evidence reveals that walking velocity affects spatio-temporal and kinematic variability as well as influences local dynamic stability in ap and ml directions [3, 24]. It is it is said that walking with higher than preferred speed increases walking variability [3, 24, 25]. Previous authors reported that walking speed has also significant influence on the complexity of plantar pressure patterns and center of pressure fluctuations [21, 30]. Liau et al. [21] reported that at the first 10 min of treadmill walking participants revealed lower COP complexity while walking with higher speed. Our results seem to be partially in conflict with this finding. A possible explanation to that discrepancy is that in previous work [21] authors did not divide COP signal to its directional subcomponents and analyzed signal fluctuations of one foot, hence only support phase of one side was analyzed. In contrast, Huijben et al. [15] reported decreased regularity in the mediolateral direction while walking faster, thus current results confirmed the validity of using detrending method proposed in the current research.

The regularity of COPap in four processing methods and vGRF in both processing methods in the current study revealed consistent entropy characteristics. In both evaluated parameters regularity increased during walking with maximum tolerated speed.

A more regular movement, with lower entropy level is a more probable and less erratic, suggesting that it is strongly controlled by a neuromuscular system, however at the same time it is less capable to make flexible adaptations to internal or external perturbations, hence it is less stable [6, 12, 21, 31]. In current experiment changes in signal regularity would suggest that in mediolateral direction increasing walking speed induced more random, thus more flexible intra-stride gait pattern. At the same time, in the anterio-posterior direction, higher walking velocity brought more regular and less erratic COP signal. Our results seem to be in line with research on changes in walking stability with increasing walking speed. Bruijn et al. [25] also reported different effects of walking speed on walking stability in ap and ml directions. Authors reported higher local stability (long-term divergence exponent) of trunk movement in ml direction with increasing walking speed, while for ap direction they observed opposite effect. Authors suggested that from stability and control perspective movements in ml direction are more important than in ap direction, due to the smaller base of support.

Recently, authors [12] have raised very important question about biological relevance of the parameter m. In our understanding there is possible solution to proper selection of the length of the template vector. In previous work, Giakas et al. [32] reported that, in human locomotion, frequencies that occur during walking are about 16 Hz for vertical and anterio-posterior and about 24 Hz for medio-lateral ground reaction forces. Stergiou et al. [33] revealed similar results. Thus, it would mean that the change in a time-series which emerge in a time interval lasting longer than 0.063 s would reflect modification in a neuromotor or mechanical state of a system. As our participants walked on motorized treadmill, we did additional FFT analysis to confirm frequencies in our data (Additional file 1: Fig. S10). In the current study m = 6 and m + 1 was equivalent to 6–7% of a stride. As mean stride time was 1.06 s, thus, 6–7% would correspond to a minimal time interval (0.064–0.074 s) which can reflect change caused by response of a neuromuscular system to more demanding walking conditions (with maximum tolerated speed). Therefore, the length of the m = 6 was valid from the biological perspective.

One potential limitation of the current study was that subjects walked on a motorized treadmill. Although treadmill walking is an efficient method to collect data from many consecutive strides, previous studies have shown that treadmills may artificially reduce the natural variability and increase dynamic stability [34]. Thus, a direct comparison of our results with results from the overground walking is unfeasible. Nevertheless, because this study quantified SampEn for parameters assessed on the same treadmill, comparisons between walking conditions remain valid. What is more, our results shows that SampEn can be promising tool for comparing walking regularity in different conditions.

In conclusion, the current study demonstrated that SampEn was able to discriminate between different walking conditions in all analyzed parameters. During walking in more demanding conditions (with maximum speed) regularity of the walking pattern expressed in the COPml signal decreased and in the COPap and vGRF increased. However, in the COPml only raw data and data detrended by our method were sensitive enough to reveal significant differences.

The results also demonstrated that for COP parameters SampEn was susceptible in the opposite way for parameter m and different preprocessing methods. The level of signal regularity or complexity may be influenced by a combination of several factors, thus authors must be extremely cautious when generalizing their findings. For future studies evaluating influence of walking velocity and using COP with its directional subcomponents and vGRF signal during treadmill walking it is advised to use raw time series, without spatial and temporal normalization. Furthermore, to maintain template vector which represents biological relevance it is advised to detect highest frequencies present in analyzed signals and evaluate minimal time interval which can reflect change caused by response of a neuromuscular system. During evaluating treadmill walking measured with 100 Hz sampling frequency it is recommended to adopt m from 6 to 10, when average stride time is up to 1 s.

## Supplementary Information

**Additional file 1.** Influence of r on SampEn, relative error analysis, Power Spectrum Density of the COPap, COPml, vGRF.

## Data Availability

The datasets supporting the conclusions of this article are included within the article and its additional files (Additional file 1), the data can be accessed through the Open ICPSR data repository service at https://doi.org/10.3886/E178941V1.
