# Supplementary material for: The influence of walking speed and effects of signal processing methods on the level of human gait regularity during treadmill walking
Source: BMC Sports Sci Med Rehabil. 2022 Dec 10;14:209. doi: 10.1186/s13102-022-00600-4 (PMC9741790; doi:10.1186/s13102-022-00600-4)
Supplement: Supplementary file 1 — Additional file 1. Influence of r on SampEn, relative error analysis, Power Spectrum Density of the COPap, COPml, vGRF. [file 13102_2022_600_MOESM1_ESM.pdf]

**Supporting information - Influence of r on SampEn, relative error analysis, Power Spectrum Density of the COPap, COPml, vGRF**

## Introduction

The aim of this supplementary material was to investigate the influence of different r level on SampEn in each variable (COPml, COPap, vGRF) and each signal processing method (WHOLE, SEGM, ZERO, NORM) in different walking conditions (Vpref, Vmax). The present analyses included combination of previously selected m=6 and different r = 0.1, 0.15, 0.2, 0.25 and 0.3. The methodological procedure was the same as the main manuscript.

Two-way ANOVA was used to compare the effect of tolerance level (r=0.1, 0.15, 0.2, 0.25, 0.3) and data processing method (Type=WHOLE, SEGM, NORM, ZERO) on the calculated sample entropy. For vertical ground reaction force (Fy) 'Type' factor had only two levels (WHOLE, SEGM). All analyses were done with previously selected vector length m=6.

In most cases parameters were normally distributed, in 6 (for 100) subgroups the normality assumption was violated, however skewness in this groups was about │0.5│ with two exceptions (r=0.1 COPap_Vmax_NORM and r=0.3 COPml_Vpref_NORM, accordingly skewness was equal 1 and 2.11). In r=0.2 all data were normally distributed. The F-test is said to be robust with respect to the assumption of normality and equality of variances so long as each group contains the same number of scores (Lindman 1974, Box 1954a,b), however, if assumption of homogeneity was violated the correlations between means and variances were inspected. The assumption of sphericity was assessed using Mauchly's test. When the assumption of uniformity was violated, an adjustment to the degrees of freedom of the F-ratio was made using Greenhouse-Geisser Epsilon, thereby making the F-test more conservative.

Consistent with results obtained by previous authors (i.e. Supplementary materials, Raffalt et al. 2019) there was an overall significant effect of tolerance level on the SampEn for all parameters in both walking conditions (Vpref, Vmax) ($p < 0.001$) (Fig. A1-A6).

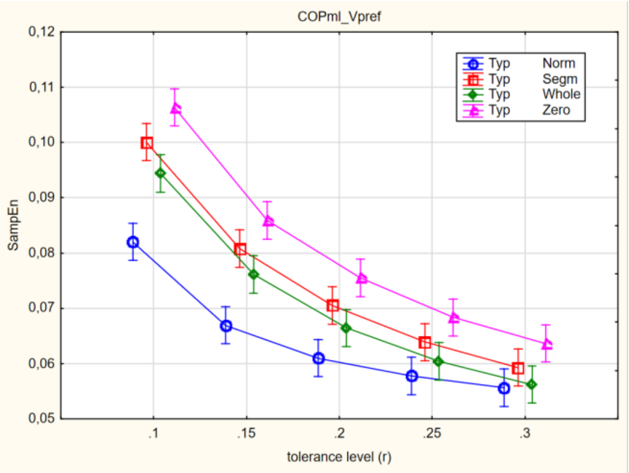


**Fig.A1** Effect of changing r on SampEn for different processing methods of COPml signal in preferred speed walking condition at m = 6. The error bars reflect the 95% confidence interval.

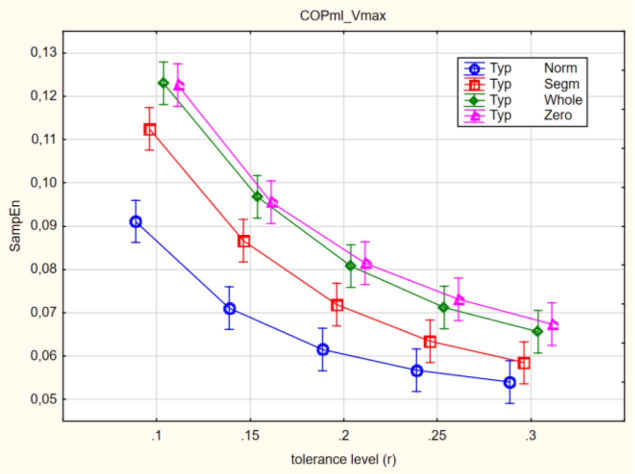


**Fig.A2** Effect of changing r on SampEn for different processing methods of COPml signal in maximum speed walking condition at m = 6. The error bars reflect the 95% confidence interval.

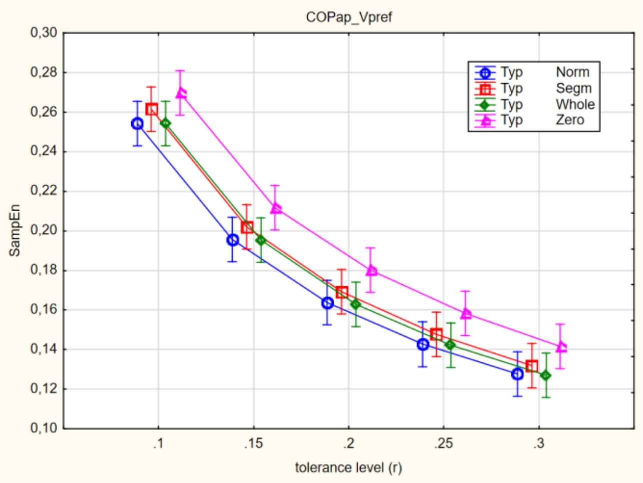


**Fig.A3** Effect of changing r on SampEn for different processing methods of COPap signal in preferred speed walking condition at m = 6. The error bars reflect the 95%confidence interval.

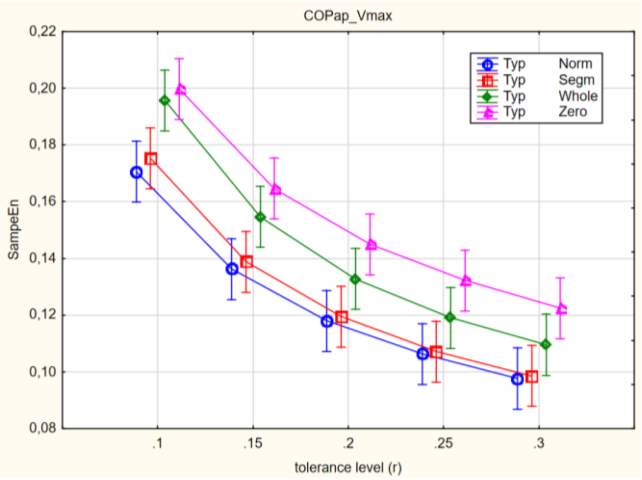


**Fig.A4** Effect of changing r on SampEn for different processing methods of COPap signal in maximum speed walking condition at m = 6. The error bars reflect the 95% confidence interval.

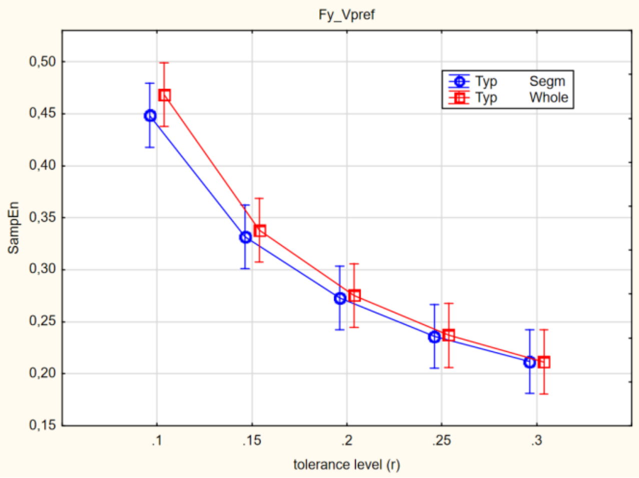


**Fig.A5** Effect of changing r on SampEn for different processing methods of vertical ground reaction force signal in preferred speed walking condition at m = 6. The error bars reflect the 95% confidence interval.

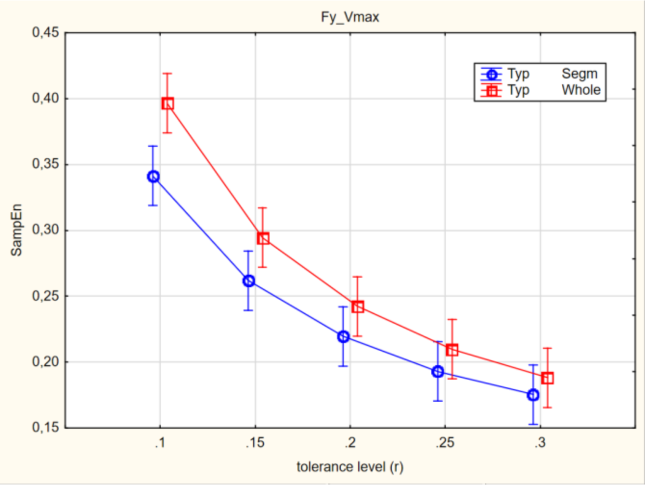


**Fig.A6** Effect of changing r on SampEn for different processing methods of vertical ground reaction force signal in maximum speed walking condition at m = 6. The error bars reflect the 95% confidence interval.

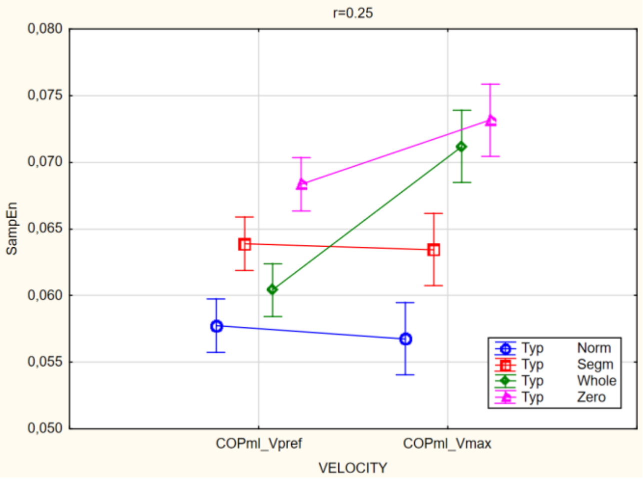


**Fig.A7** Effect of changing walking velocity on SampEn for different processing methods of COPml at m = 6 and r=0.25. The error bars reflect the 95% confidence interval.

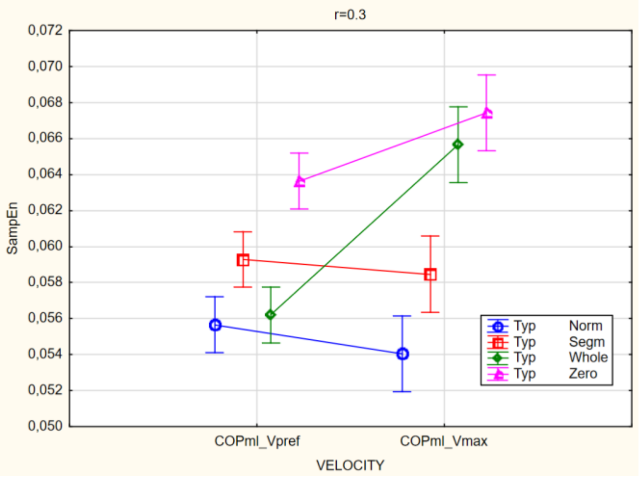


**Fig.A8** Effect of changing walking velocity on SampEn for different processing methods of COPml at m = 6 and r=0.3. The error bars reflect the 95% confidence interval.

The results are not surprising, there was an overall tendency to a lower SampEn across the Fy and COPap/ml parameters with increasing r-values (more relaxed criteria for counting similar vectors). Results indicate that relations between signal types did not change with different r (Fig.3-6). Only for COPml in both walking conditions (Vpref and Vmax) there was a significant interaction Type*r effect (p <0.001)(Fig.1,2). In COPml, in Vpref condition, the post-hoc tests revealed that the relation between 'Types' differed with increasing r. In r=0.2 signal ZERO and WHOLE varied significantly and this difference disappeared in r=0.25 and r=0.3. The same happened with NORM and SEGM signal (Fig.1). Similar situation could be seen between r=0.2 and r=0.3 in COPml_Vmax (Fig.2).

To check whether discriminatory abilities of SampEn in COPml remained the same in r=0.25 and r=0.3 as in r=0.2, we conducted One-Way ANOVA with Repeated Measures. Post-hoc analysis revealed that in r=0.25 and r=0.3 only signal ZERO and

WHOLE increased significantly during walking with maximum speed when compared to preferred speed ($p<0.01$)(Fig. 7,8). These results are consistent with outcomes obtained with r=0.2.

Results of aforementioned analysis indicates that using r=0.2 with our data is valid.

To check whether our parameters selection (m=6 and r=0.2) represents the acceptable length of the Confidence Interval (CI) around the SampEn, we have adopted method proposed by Lake et al. (2002), which penalizes conditional probability near 0 and near 1. Figure 9 shows that for m = 6 and r=0.2 the CI did not exceed the ~10% of the SampEn (maximum relative error no higher than ~0.023)

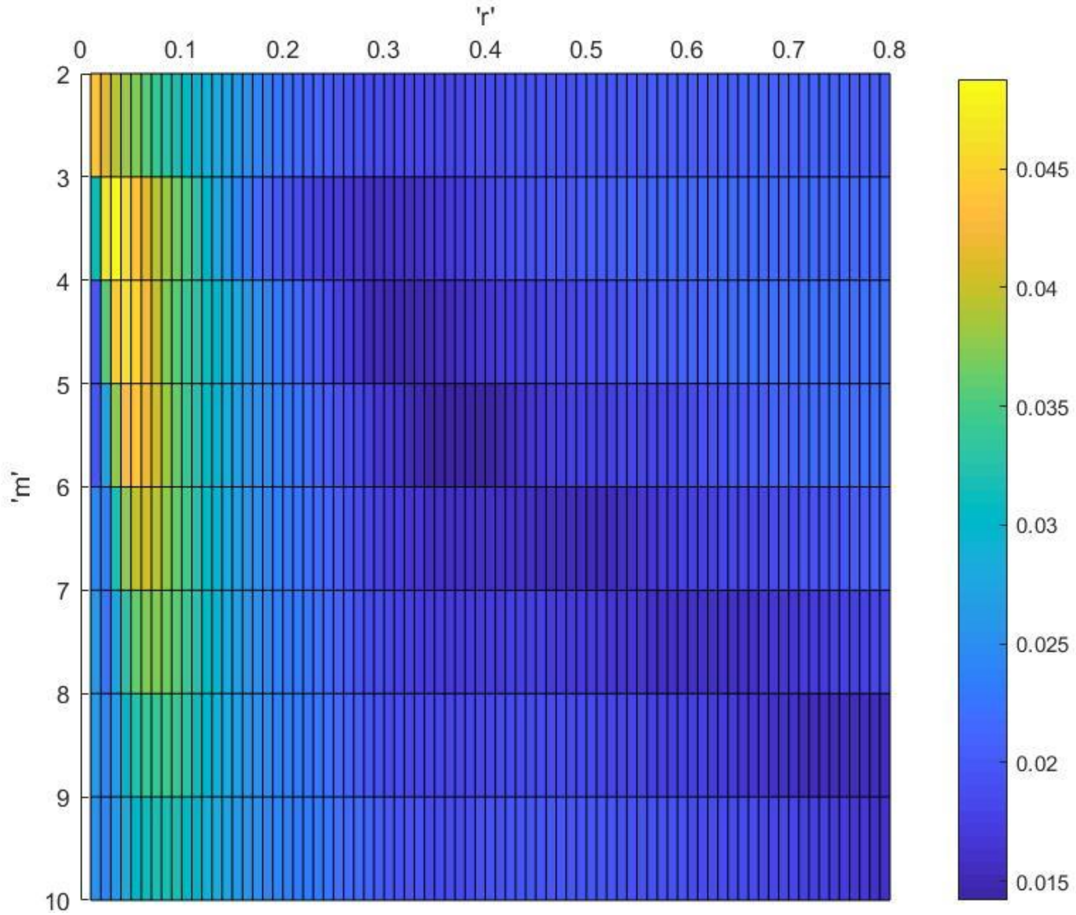


**Fig.A9** The mean value of a sample entropy (SampEn) efficiency metric for 23 COPap data records, schemed as a function of m and r. A value of 0.023 corresponds to a 95% confidence interval (CI) that is less than 10% of the SampEn estimate.

As it can be seen, there are several combinations of m and r which give acceptable level of the SampEn relative error.

The results of the power spectral density (PSD) of the vertical ground reaction force (vGRF), center of pressure in mediolatreal direction (COPml) and anteroposterior direction (COPap) for 23 participants during walking on the treadmill with preferred walking speed.

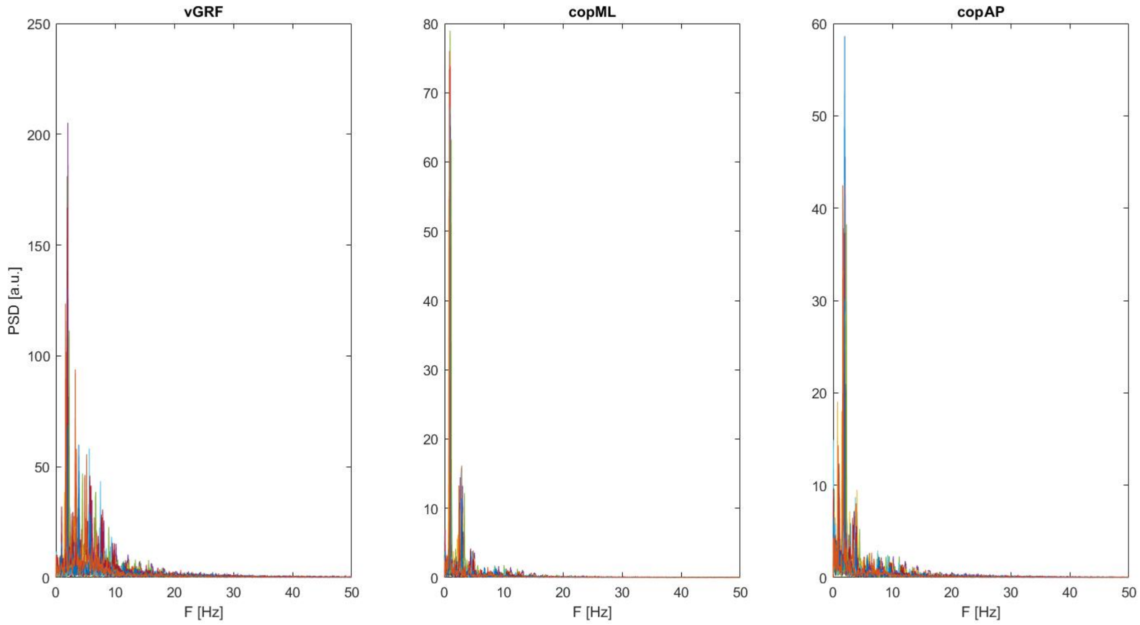


**Fig.A10 Power spectral density**
